# Classification of nasal polyps and inverted papillomas using CT-based radiomics

**DOI:** 10.1186/s13244-023-01536-0

**Published:** 2023-11-13

**Authors:** Mengqi Guo, Xuefeng Zang, Wenting Fu, Haoyi Yan, Xiangyuan Bao, Tong Li, Jianping Qiao

**Affiliations:** 1 School of Physics and Electronics, Shandong Normal University, No. 88, Wenhua East Road, Lixia District, Jinan, Shandong 250014 China; 2 Department of Radiology, Shandong First Medical University & Shandong Academy of Medical Sciences, No.619 Chang Cheng Road, Daiyue District, Taian, 271016 Shandong China; 3 Department of Radiology, Shandong Provincial Hospital Affiliated to Shandong First Medical University, No.324 Jingwuwei 7Th Road, Huaiyin District, Jinan, Shandong 250021 China

**Keywords:** Inverted papilloma, Nasal polyp, Computed tomography, Radiomics

## Abstract

**Objectives:**

Nasal polyp (NP) and inverted papilloma (IP) are two common types of nasal masses. And their differentiation is essential for determining optimal surgical strategies and predicting outcomes. Thus, we aimed to develop several radiomic models to differentiate them based on computed tomography (CT)-extracted radiomic features.

**Methods:**

A total of 296 patients with nasal polyps or papillomas were enrolled in our study. Radiomics features were extracted from non-contrast CT images. For feature selection, three methods including Boruta, random forest, and correlation coefficient were used. We choose three models, namely SVM, naive Bayes, and XGBoost, to perform binary classification on the selected features. And the data was validated with tenfold cross-validation. Then, the performance was assessed by receiver operator characteristic (ROC) curve and related parameters.

**Results:**

In this study, the performance ability of the models was in the following order: XGBoost > SVM > Naive Bayes. And the XGBoost model showed excellent AUC performance at 0.922, 0.9078, 0.9184, and 0.9141 under four conditions (no feature selection, Boruta, random forest, and correlation coefficient).

**Conclusions:**

We demonstrated that CT-based radiomics plays a crucial role in distinguishing IP from NP. It can provide added diagnostic value by distinguishing benign nasal lesions and reducing the need for invasive diagnostic procedures and may play a vital role in guiding personalized treatment strategies and developing optimal therapies.

**Critical relevance statement:**

Based on the extraction of radiomic features of tumor regions from non-contrast CT, optimized by radiomics to achieve non-invasive classification of IP and NP which provide support for respective therapy of IP and NP.

**Key points:**

• CT images are commonly used to diagnose IP and NP.

• Radiomics excels in feature extraction and analysis.

• CT-based radiomics can be applied to distinguish IP from NP.

• Use multiple feature selection methods and classifier models.

• Derived from real clinical cases with abundant data.

**Graphical Abstract:**

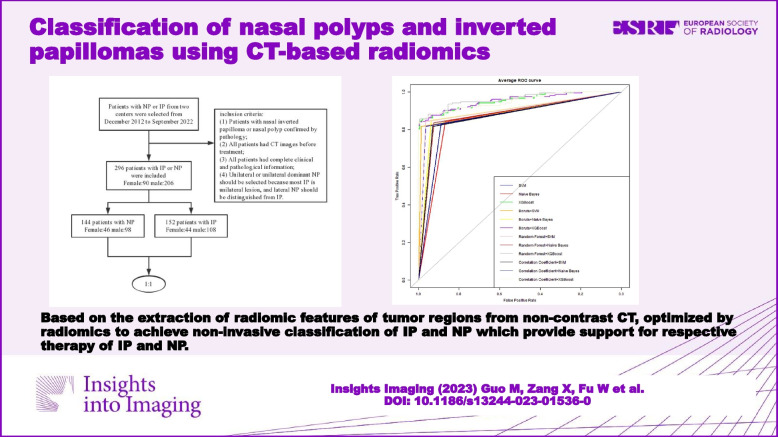

## Introduction

Sinonasal inverted papilloma (IP) is a nonmalignant disease that primarily affects the paranasal sinuses and nasal cavity [1]. It has garnered significant clinical attention due to its high recurrence rate and potential to progress into squamous cell carcinoma [2, 3]. For instance, S MIRZA et al. reviewed sixty-three case series with adequate data, finding 163 (7.1%) cases of synchronous carcinoma out of 2297 cases and 74 (3.6%) cases of metachronous carcinoma out of 2047 cases [4]. Among these synchronous carcinomas, the vast majority were squamous cell carcinomas. And some cases of transitional cell carcinoma, adenocarcinoma, mucoepidermoid carcinoma, and verrucous carcinoma were also found. However, in clinical practice, IP is often mistaken for a common inflammatory lesion called nasal polyp, causing confusion among clinicians. Nasal polyps (NP) are benign masses associated with chronic rhinosinusitis originating from the nasal cavity and sinuses’ mucosa. Current clinical guidelines frequently classify nasal polyps as a subtype of chronic rhinosinusitis, with a diagnosis established when nasal symptoms persist for over 3 months and polyps are observed in the nasal cavity [5]. NP treatment predominantly involves conservative treatment including corticosteroids or minimally invasive surgery, while endoscopic surgery is considered the primary approach for IP therapy, involving wide resection of the affected mucosa and bone [6]. Consequently, distinguishing between IP and NP is crucial for determining the optimal surgical approach and subsequent treatment.

Clinically, nasal congestion, rhinorrhea, and decreased olfactory function are commonly reported symptoms in nonmalignant lesions of the nasal sinuses and cavity [7]. These symptoms can be observed in both IP and NP. While nasal polyps typically occur on both sides of the nasal cavity, the presence of polyps limited to one side should raise suspicion of IP. Therefore, clinical symptoms alone have limited diagnostic value in distinguishing between IP and NP. Additionally, preoperative biopsy is often considered an essential method for determining the nature of nasal tumors. However, this approach can be challenging to execute, especially in certain hospitals that lack access to high-quality pathological support. Furthermore, the biopsy procedure itself carries risks such as tissue crushing from instruments like forceps, increased bleeding, and structural damage when dealing with deep lesions or performing incisions under general anesthesia. Moreover, the growth of NK/T-cell lymphomas can lead to vascular occlusion and extensive tissue necrosis, making it difficult to obtain sufficient tumor tissue [8]. All of these factors may result in an inadequate representation of the collected tissues. Moreover, the diagnosis of nasal tumors is sometimes subjective and relies on visual assessment, which can vary depending on the device used and the operator’s experience [9]. Therefore, relying solely on clinical symptoms and preoperative biopsy for distinguishing between IP and unilateral NP can be unreliable and problematic.

Preoperative imaging examination is commonly used to assess nasal tumors. Computed tomography (CT) is the best modality for assessing bone characteristics, such as erosion, proliferative sclerosis, destruction, and calcification within lesions. It can provide some radiological diagnosis based on focal hyperostosis, although it may still be non-specific and inadequate for precisely locating the origin and extent of the tumor [10]. On the other hand, magnetic resonance imaging (MRI), which usually serves as a complementary diagnostic method to CT, provides excellent clinical results for evaluating the soft tissue components of tumors and assessing tumor infiltration beyond the bone. It is a suitable tool for distinguishing nasal lesions, especially T2-weighted images, and has been recommended as the preferred imaging method recently [11]. To go a step further, the accuracy of CT in diagnosing inverted papilloma of the nasal cavity is high, but it lacks specific diagnostic criteria [10]. On CT images, it typically appears as a soft tissue density mass within the unilateral nasal cavity or sinus, exhibiting polypoid expansile growth. The maxillary sinus, lateral nasal wall, and ethmoid sinus are common locations, with characteristic bone proliferation or resorption, a sign referred to as “osteitis sign.” CT also reveals polypoid masses originating from the lateral nasal wall, nasal roof, and ethmoid sinus and involving the nasal cavity and sinuses, indicating nasal polyps. In cases where differentiation from tumors is challenging, enhanced CT or MRI is necessary to aid in diagnosis. In fact, enhanced MRI scans often show a typical labyrinthine or convoluted cerebriform pattern (CCP), suggesting inverted papilloma [2]. That is the reason why differential diagnosis of nasal polyps and inverted papillomas relies more on enhanced MRI scans. However, despite these imaging techniques, accurately determining the site of origin and differentiating from similar lesions remains challenging. Therefore, there is a need for a more efficient, computer-assisted diagnostic classification algorithm that can save time and improve accuracy. Such an algorithm would assist clinicians in making timely decisions and enable further evaluation of suspected cases.

In recent years, deep learning has emerged as a popular and highly valued topic, especially in the medical field. One notable application is radiomics, a rapidly advancing image analysis technology that plays a crucial role in precision medicine. Radiomics enables the extraction and quantification of features from medical images, providing valuable information on the heterogeneity of tumors. These radiomic features are instrumental in distinguishing diseases with similar clinical presentations and determining disease staging [12]. As a result, they greatly contribute to etiological diagnosis, management strategies, and prognostic assessment.

Recent studies have reported successful applications of convolutional neural networks (CNN) in identifying clinically significant portal hypertension using CT and MRI images [13]. Additionally, there have been indications that trained CNN models can differentiate between NP and IP using nasal endoscopic images [14]. Based on these investigations, it is reasonable to believe that CT radiomics can be applied to the diagnosis of otolaryngological diseases. CT has long been a significant diagnostic tool for nasal inverted papilloma, and existing literature suggests that features such as bone thickening, bone erosion, and relative CT numbers on CT images hold significant value in differentiating IP from NP [15].

In summary, the objective of this study is to analyze and discuss the application of CT radiomics in the classification of nasal polyps and inverted papilloma. The findings aim to provide guidance to otolaryngologists in their diagnostic process.

## Materials and methods

### Patients

**Fig. 1:**
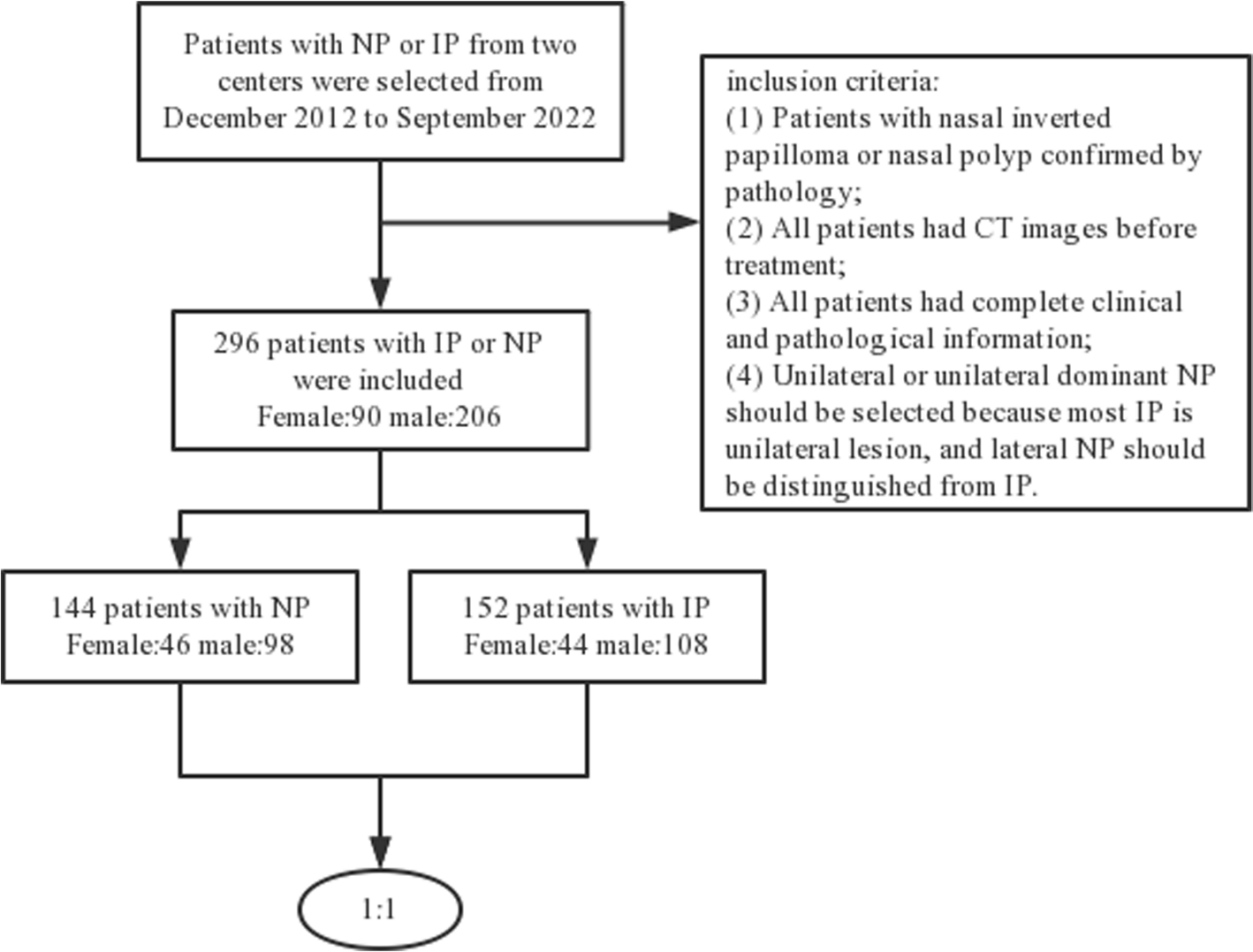
The workflow of the collection of patients. Created using WPS office

The institutional review board of Shandong First Medical University has approved our research project. Due to its retroactive nature, written informed consent is naturally waived. We retrieved the CT scan results of suspected NP or IP patients from December 2012 to September 2022 from the picture archiving and communication system (PACS) and conducted follow-up examinations. The CT images of 296 participants were gathered who met the following inclusion criteria (Fig. 1): (1) patients with nasal inverted papilloma or nasal polyp confirmed by pathology; (2) all patients had CT images before treatment; (3) all patients had complete clinical and pathological information; and (4) unilateral or unilateral dominant NP should be selected because most IP is unilateral lesion, and lateral NP should be distinguished from IP. Unilateral dominant NP (bilateral polyps, but most of them are lateral) is acknowledged when it fits the following requirements: in one side of the nasal cavity, NP reaches the conjoint nasal cavity, and it is located in the middle nasal meatus on the other side. The exclusion criteria are as follows: (1) lesions were small in size and could not extract sufficient imaging features; (2) poor image quality or heavy artifacts; (3) when bilateral NP does not show laterality, such as bilateral NP reaching the conjoint nasal cavity or bilateral NP in the middle nasal meatus, we will put it out from the study. The resulting workflow is shown in Fig. 1.

### Image acquisition

This was a multi-center study that included CT examinations without contrast media. We collected these relevant data from the two medical centers of the hospital affiliated with our school.

In two centers, patients underwent CT scanning using multidetector-row CT systems (Somatom Force, Siemens Healthcare; Discovery 750, GE Healthcare and Ingenuity CT, Philips; Aquilion ONE, TOSHIBA; Somatom Force, Siemens Healthcare; Lispeed 64, GE Healthcare) respectively. We described the scanning by following parameters: set the voltage to 100 kV or 120 kV; the tube current (automatic modulation tube current was used) was 100–400 mAs, and CTDIvol was 15–60 mGy; a matrix of 512 × 512, and a pitch of 0.6 or 1.0; detector collimation was installed to 192 × 0.6 mm or 64 × 0.625 mm; axial images with 1 mm slice thickness were reconstructed.

### Image preprocessing

**Fig. 2:**
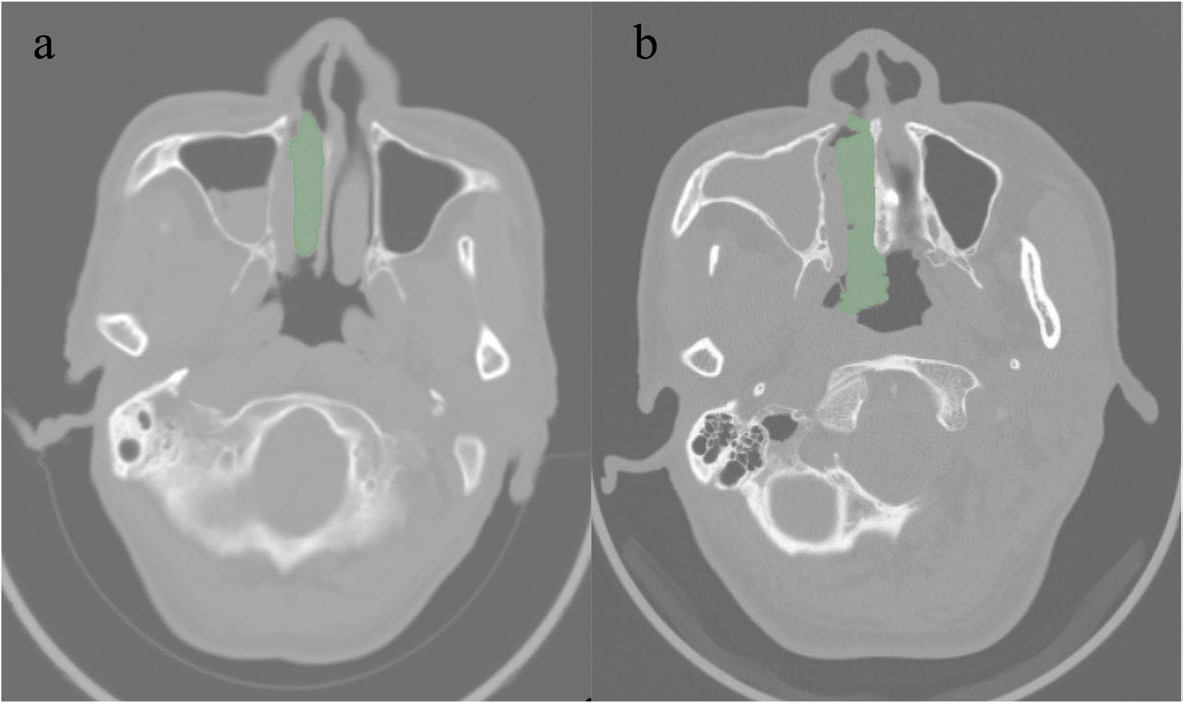
Handcrafted annotation CT images of IP and NP. **a** IP; **b** NP. Axial noncontrast CT scans of the paranasal sinuses demonstrate a mass filling the right nasal cavity and extending into the maxillary sinus. **a** The right middle nasal cavity is filled with soft tissue-like density lesion; the right middle nasal meatus is enlarged, and the right middle and lower turbinates are displaced. The right maxillary sinus and nasal mucosa are thickened, and a little gas density shadow can be seen; the nasal septum is partially deflected to the left. **b** Soft tissue density lesion filling can be seen in the right maxillary sinus and nasal meatus; the nasal meatus is narrow, and the lesion protrudes backward to the posterior naris. No significant absorption or destruction of the bone in the residual sinus wall is observed; the left maxillary sinus is well gasified, and the sinus cavity is clearly displayed; the nasal septum is in the middle

The noncontrast and thin slice images were downloaded from PACS and stored in DICOM format. And we resampled the CT image to 1 × 1 × 1, which means that the sampling interval is 1 mm on all three axes (*x*-axis, *y*-axis, and *z*-axis, respectively). This means that each two adjacent pixels in the image are separated by 1 mm in each direction. Then, we imported the images into the Slicer 5.0.3 software, and the ROIs for the 3D images were subsequently confirmed layer-by-layer by two otolaryngology radiologists. In this process, we found the layer with the largest tumor volume displayed and outlined this layer and its adjacent upper and lower layers, totaling five layers. The relevant information for the lesion dataset and manually annotated CT images is shown in Table 1 and Fig. 2.

**Table 1:** Image data of patients

	Patients	Lesions	Images
NP	144	144	720
IP	152	152	760
Total	296	296	1480

*Lesions* NP and IP, *Images* handcraft-annotation CT images, *NP* nasal polyp, *IP* inverted papilloma

### Feature extraction

The ROIs delineated by the otolaryngologist were used as mask files, and the CT images obtained after resampling of the original CT images were used as image files. The mask and image files were imported into Python, and 1288 features were extracted using radiomics.

### Feature selection

Not all features are beneficial for model classification, so feature selection is necessary. For feature selection, three methods including Boruta, random forest, and correlation coefficient were used. From 1288 features, they selected 96, 73, and 257, respectively.

### Model construction

We choose three models, namely SVM, naive Bayes, and XGBoost, to perform binary classification on the selected features. And the data was validated with 10-fold cross-validation. Nine results were obtained by combining three feature selection methods and three models.

### Statistical analysis

**Fig. 3:**
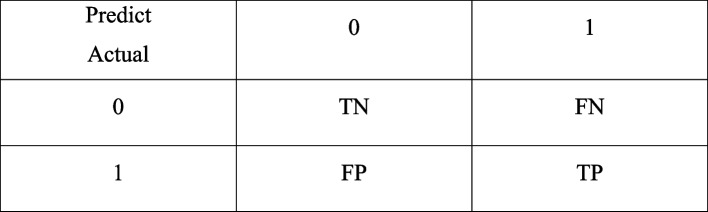
Confusion matrix

The receiver operator characteristic (ROC) curve is constantly regarded as a graphics technology to describe and evaluate the accuracy and sensitivity as well as specificity of diagnostic tests or models. In this study, we generated ROC curves to compare the accuracy of different classifiers. The confusion matrix was shown in Fig. 3. We selected AUC, accuracy, sensitivity, specificity, precision, and F1 score to analyze the data in this paper.

## Results

### Demographic characteristics

We retrospectively reviewed 1480 thin slice-thick images of 296 subjects with pathologically confirmed lesions of IP or NP at our hospital. Of the 296 patients, 152 had IP, and 144 had NP. Table 2 aggregates the demographic characteristics of the patients in our paper. From the table, there are few significant differences in the percentage sex ratio between the two groups.

**Table 2:** Demographic of study subjects

	IP (*n* = 152)	NP (*n* = 144)	*p*
Age mean ± SD (range)	41.00 ± 18.86 (15–82 years)	53.44 ± 13.50 (6–83 years)	
Gender			0.575
Male	108 (71.1)	98 (68.1)	
Female	44 (28.9)	46 (31.9)	

### Visualization of feature selection

**Fig. 4:**
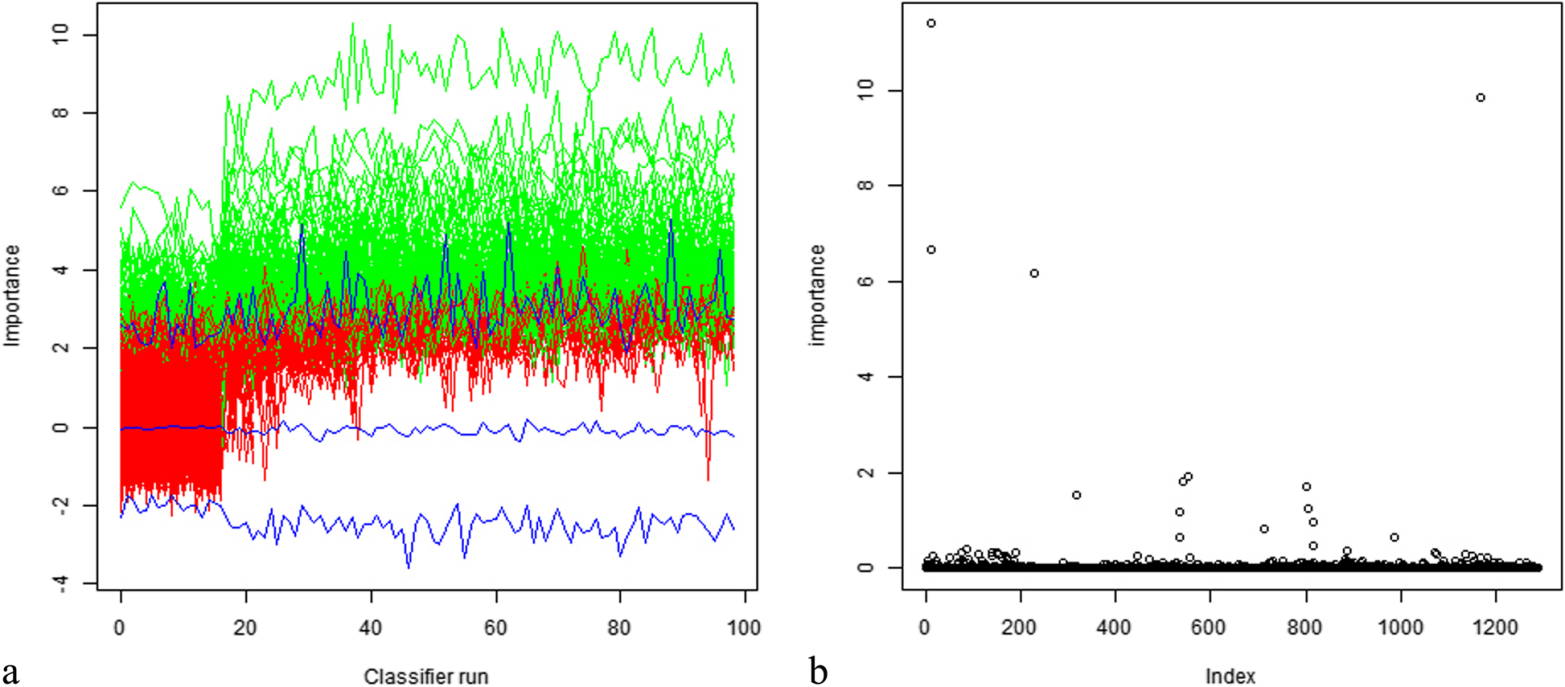
Visual representation of Boruta and random forest

**Fig. 5:**
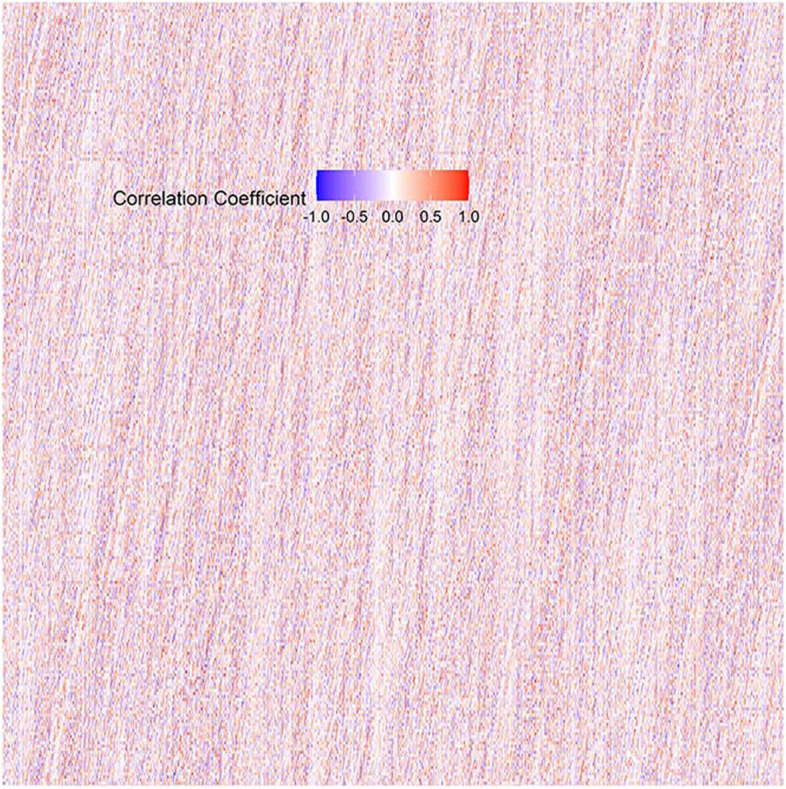
Visual representation of correlation coefficient

In this study, 1288 features were extracted, and three methods of feature selection (Boruta, random forest, and correlation coefficient) were applied. The extracted image radiomics features were divided into the following three main categories: second-order and higher-order texture-based features, first-order gray-scale histogram features, and shape features. Figures 4 and 5 provide a visual representation of these methods.

### Diagnostic performance of various classifier models

**Fig. 6:**
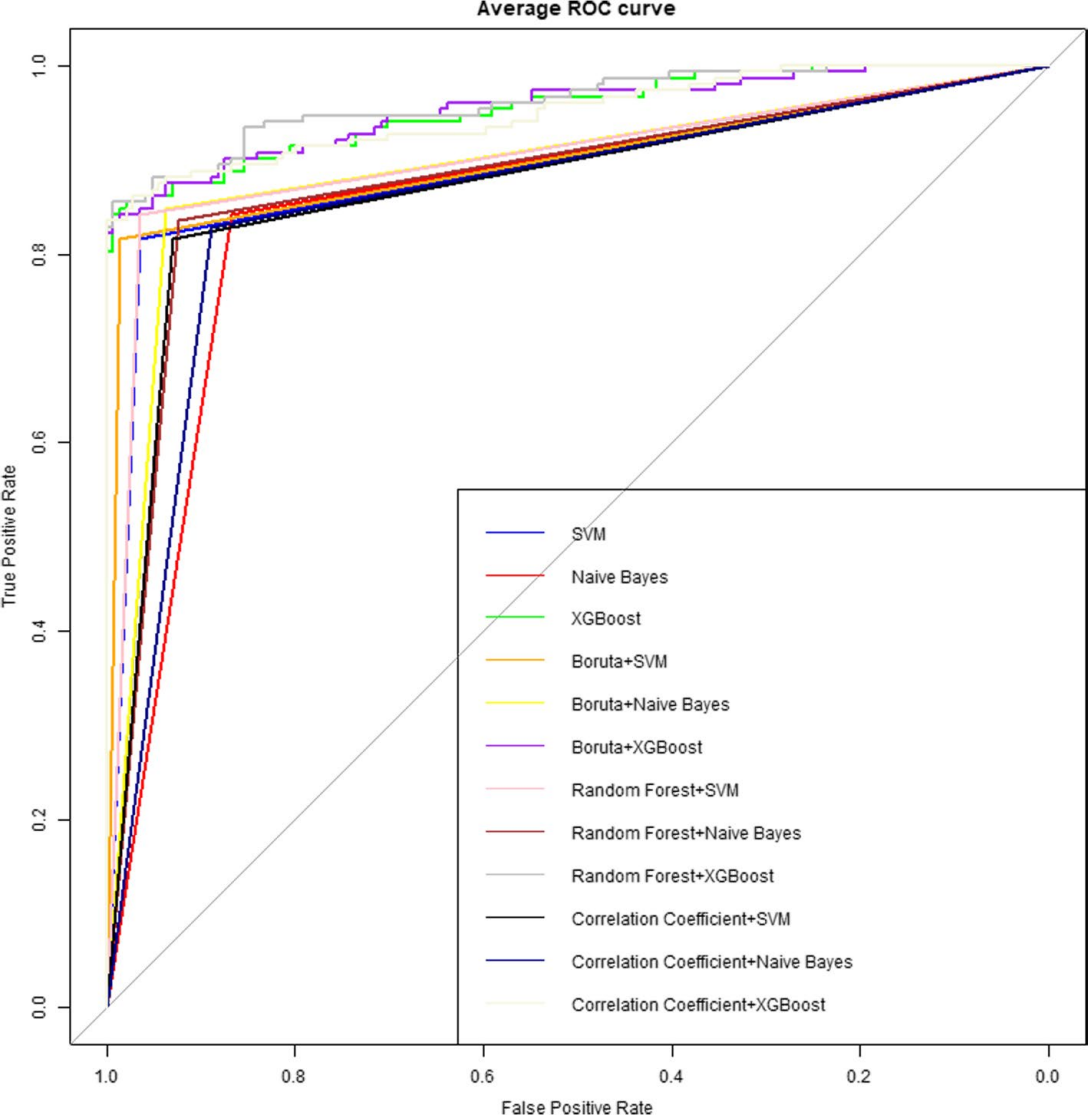
ROC of the 3 types of machine learning models under various feature selection methods

We applied several different classifier models to the differential diagnosis of NP and IP, and then obtained the results shown in Table 3. According to Table 3, the highest specificity (0.9929), sensitivity (0.8896), accuracy (0.9153), and AUC (0.922) are displayed. The superb AUC performance of 0.922, 0.9078, 0.9184, and 0.9141 were shown for the XGBoost model, compared to the normal performance of the other models. Consequently, the performance order of these classifiers is as follows in our research: XGBoost > SVM > Naive Bayes. We summarized the ROC equipped with these radiomic models with high AUC under various feature selection methods, as shown in Fig. 6. Finally, we obtained with high AUC under different feature selection methods. In addition, diagnostic performance of the radiomics models in independent test CT scans was shown in Table 4.

**Table 3:** Average performance of various machine learning models

Feature selection	Model name	AUC	Accuracy	Sensitivity	Precision	Specificity	F1 score
No	SVM	0.8969	0.8883	0.8270	0.9644	0.9668	0.8842
	Naive Bayes	0.8604	0.8540	0.8543	0.8724	0.8666	0.8574
	XGBoost	0.9220	0.9153	0.8510	0.9929	0.9929	0.9143
Boruta	SVM	0.9068	0.8984	0.8260	0.9838	0.9876	0.8938
	Naive Bayes	0.9013	0.8916	0.8602	0.9368	0.9424	0.8901
	XGBoost	0.9078	0.9018	0.8573	0.9568	0.9582	0.9017
Random forest	SVM	0.9077	0.9018	0.8512	0.9644	0.9643	0.8995
	Naive Bayes	0.8872	0.8777	0.8466	0.9220	0.9278	0.8770
	XGBoost	0.9184	0.9120	0.8896	0.9443	0.9473	0.9122
Correlation coefficient	SVM	0.8803	0.8714	0.8263	0.9310	0.9342	0.8682
	Naive Bayes	0.8659	0.8576	0.8407	0.8842	0.8911	0.8567
	XGBoost	0.9141	0.9086	0.8707	0.9603	0.9575	0.9093

**Table 4:** Diagnostic performance of the radiomics models in independent test CT scans

Feature selection	Model name	Evaluation indicators	1	2	3	4	5	6	7	8	9	10
		Accuracy	0.9310	0.9333	0.8000	0.7586	0.8966	0.8966	0.9333	0.9000	0.8333	1.0000
	SVM	FN	2	2	6	7	3	3	1	1	3	0
		FP	0	0	0	0	0	0	1	2	2	0
		Accuracy	0.7931	0.9000	0.7667	0.7586	0.8276	0.8276	0.9333	0.9000	0.8667	0.9667
No	Naive Bayes	FN	2	3	6	5	3	2	1	0	2	0
		FP	4	0	1	2	2	3	1	3	2	1
		Accuracy	0.9310	0.9333	0.8333	0.8276	0.8966	0.9310	0.9667	0.9667	0.8667	1.0000
	XGBoost	FN	2	2	5	5	3	2	1	1	3	0
		FP	0	0	0	0	0	0	0	0	1	0
		Accuracy	0.9310	0.8667	0.8000	0.7931	0.8966	0.8966	0.9667	0.9333	0.9000	1.0000
	SVM	FN	2	3	6	6	3	3	1	1	3	0
		FP	0	1	0	0	0	0	0	1	0	0
		Accuracy	0.8966	0.9000	0.8000	0.8276	0.8966	0.8621	0.9667	0.9000	0.8667	1.0000
Boruta	Naive Bayes	FN	2	3	6	5	2	2	1	0	2	0
		FP	1	0	0	0	1	2	0	3	2	0
		Accuracy	0.9310	0.9333	0.8000	0.8276	0.8966	0.8966	0.9333	0.9333	0.8667	1.0000
	XGBoost	FN	2	2	5	5	3	2	1	1	2	0
		FP	0	0	1	0	0	1	1	1	2	0
		Accuracy	0.9655	0.9333	0.8000	0.8621	0.8276	0.8966	0.9333	0.9333	0.8667	1.0000
	SVM	FN	0	2	6	4	4	3	1	1	3	0
		FP	1	0	0	0	1	0	1	1	1	0
		Accuracy	0.8966	0.9000	0.8333	0.8276	0.7931	0.7931	0.9667	0.9000	0.8667	1.0000
Random forest	Naive Bayes	FN	2	3	5	5	4	3	1	0	2	0
		FP	1	0	0	0	2	3	0	3	2	0
		Accuracy	0.8966	0.9333	0.8333	0.8621	0.8966	0.9310	0.9667	0.9000	0.9000	1.0000
	XGBoost	FN	1	2	5	4	2	1	1	1	1	0
		FP	2	0	0	0	1	1	0	2	2	0
		Accuracy	0.8966	0.9000	0.8000	0.7931	0.8276	0.8966	0.9333	0.8333	0.8333	1.0000
	SVM	FN	2	2	6	6	4	3	1	1	3	0
		FP	1	1	0	0	1	0	1	4	2	0
		Accuracy	0.8621	0.9000	0.7667	0.7931	0.8276	0.7931	0.9333	0.9333	0.8333	0.9333
Correlation coefficient	Naive Bayes	FN	2	2	6	5	4	3	1	1	3	0
		FP	2	1	1	1	1	3	1	1	2	2
		Accuracy	0.9655	0.9333	0.8333	0.8276	0.8966	0.8966	0.9000	0.9333	0.9000	1.0000
	XGBoost	FN	1	2	5	5	3	2	1	1	1	0
		FP	0	0	0	0	0	1	2	1	2	0

## Discussion

Accurate differentiation between sinonasal IP and NP has always been a significant challenge in clinical practice. The resemblance in clinical symptoms and the results of various auxiliary examinations contribute to the difficulty in distinguishing between these conditions [16]. Given that IP typically requires more extensive surgical resection, whereas NP can be managed with medical treatment or minimally invasive surgery, accurate preoperative prediction of IP or NP is crucial for determining the optimal surgical strategy and predicting the prognosis.

In the field of otolaryngology, clinicians commonly employ CT scans prior to surgery to assess the extent of IP involvement in the sinuses and determine the appropriate surgical strategy. Earlier studies have indicated that focal hyperostosis is a notable CT feature for predicting IP [17]. For instance, Glikson et al. conducted a study involving 195 IP patients and discovered that 65% of them exhibited focal hyperostosis [18]. However, the study concluded that the presence of focal hyperostosis detected in preoperative CT scans did not significantly impact the long-term prognosis of inverted papilloma resection. These findings collectively suggest that relying solely on the presence of focal hyperostosis is insufficient for distinguishing between IP and NP. Another study by Sukenik et al. reported a specificity of only 20% for preoperative CT examination in evaluating IP [19]. Similarly, in our own study, a considerable number of IP patients did not exhibit focal hyperostosis. These research findings highlight the insufficiency of focal hyperostosis alone in differentiating IP from NP.

Nevertheless, the potential of CT in distinguishing the two remains to be tapped. Sano et al. tried to evaluate the meaning of relative CT value (CT attenuation number relative to those of the brainstem) for distinguishing NP and IP [15]. According to this research, CT values of the NP set and IP set were 28.0 ± 12.8 HU and 38.0 ± 10.0 HU separately, suggesting IP had superior CT values than NP. Therefore, we could acquire a simple and creative reference parameter for diagnosis of NP and IP. Based on this, our study tries to find a new way to extract image features of two diseases, including CT values, to maximize the use of CT images to achieve the identification of IP and NP, which involves the field of radiomics.

Radiomics currently plays a crucial role in non-invasive clinical diagnosis, particularly in the field of tumors, and has shown promise in classification, staging, predicting healing outcomes of various tumors, and evaluating treatment effects of different surgical options [20–25]. Moreover, radiomics can extract informative features from images that may be difficult for humans to detect [26, 27]. As the clinical application of radiomics continues to be explored, an increasing number of otorhinolaryngology practitioners have started applying radiomics to their field. Yan et al. demonstrated that a model combining morphological features and MR radiomics could accurately predict inverted papilloma with squamous cell carcinoma transformation, potentially enhancing patient consultation and facilitating more precise treatment planning [28]. Studies have also shown the feasibility of using artificial intelligence and radiomics to diagnose difficult cases involving small round cell malignant tumors (SRCMTs) or non-SRCMTs [29, 30]. These findings suggest the promising potential of non-invasive methods in clinical practice. Recent research by Du et al. revealed that a novel combination of clinical features and MRI-based radiomics could effectively differentiate between IP and NP invading the olfactory nerve, highlighting the potential of radiomic models in addressing nasal diseases [31].

Radiomics has demonstrated strong capabilities in feature extraction and texture analysis, making it suitable for the intrinsic appearance evaluation of tumors. Previous studies have shown that radiomics can provide valuable insights, particularly in cases where radiologists have limited recognition ability, enabling precise and automatic tumor extraction [32–35]. While MRI is known for its ability to display soft tissues, making it potentially more suitable for differentiating between tumors and inflammatory conditions such as IP and NP, in clinical practice, patients suspected of having IP or NP often undergo CT examinations initially due to the high cost of MRI and the limited visualization of bone structures. Therefore, this study focusing on CT-based radiomics is more practical and applicable in a clinical setting compared to MRI-based radiomic research.

In our study, we developed several radiomic models based on CT features to distinguish between IP and NP. The results demonstrated that these radiomic models, particularly the XGBoost classifier, exhibited excellent diagnostic accuracy, confirming their clinical application value. Our study possesses several advantages compared to previous studies on radiomics models for differentiating NP and IP. Firstly, we approached the analysis of IP and NP from a clinical perspective rather than relying solely on a database, and our study included a larger population compared to most relevant studies. Secondly, we employed three different methods of feature extraction (correlation coefficient, Boruta, and random forest), which contributed to improved diagnostic performance compared to previous radiomics models. Thirdly, we utilized multiple classifier models for classification, and some of these models demonstrated outstanding performance. Additionally, our study employed ten-fold cross-validation to validate the accuracy of the algorithm, a step that was lacking in previous studies.

Although our study has achieved some results, there are certain limitations that should be discussed. Firstly, we did not incorporate clinical factors or clinical indicator features into the radiomics analysis. Clinical diagnosis is typically based on the comprehensive evaluation of all available data in clinical practice, including imaging information of surrounding tissues and the impact of the tumor on neighboring structures. Therefore, it is important to conduct further research on clinical indicators and explore their performance and impact on the identification of IP and NP. Fusion experiments incorporating clinical indicators could enhance the diagnostic efficiency of IP.

Secondly, the number of clinical cases in our study was limited, particularly the data on IP patients, which are relatively scarce in hospitals. This limited sample size affects the generalizability of our model. It would be beneficial to collect a larger and more diverse dataset to improve the robustness and applicability of the radiomics models.

Lastly, the machine learning methods employed in our study mainly focused on traditional algorithms and did not involve deep learning techniques. Deep learning has demonstrated significant advancements and achievements in various research fields. Therefore, further investigations utilizing deep learning methods are expected to contribute to the differential diagnosis between IP and NP.

## Conclusions

In summary, we have constructed several CT-based radio models with reasonably great accuracy in distinguishing IP from NP. It not only demonstrates diagnostic value in distinguishing benign nasal lesions, thereby reducing the need for invasive diagnostic procedures, but can also be used to guide personalized treatment strategies and develop optimal therapies.

## Data Availability

The datasets analyzed during the current study are available from the corresponding author on reasonable request.

## Abbreviations

CCP: Convoluted cerebriform pattern
CNN: Convolutional neural networks
CT: Computed tomography
IP: Inverted papilloma
MRI: Magnetic resonance imaging
NP: Nasal polyp
PACS: Picture archiving and communication system
ROC: Receiver operator characteristic
SRCMTs: Small round cell malignant tumors
